# Bound Volumes

**Published:** 1877-05

**Authors:** 


### *BOUND VOLUMES.*

We will send one volume of Hall's Journal of Health, good cloth binding, containing more than 250 pages of Dr. Hall's best articles on health subjects, postage paid, on receipt of sixty cents, or two volumes for $1.10, and four volumes for $2.00; eleven volumes for $5.00. For $1.00 we will send, postpaid, one bound volume of Hall's Journal of Health, containing over 500 pages. Our readers now have an opportunity to procure Dr. Hall's best works at about the cost of binding them. Address, Hall's Journal of Health, New York.

### *RIVERS OF IMPURE BLOOD*

Flow and vibrate through the system of those tainted with Scrofula, Salt Rheum, Barber's Itch, Syphilis, Eruptions, or Pimples on the Face, Roughness or Redness of the Skin, etc. Stafford's Iron and Sulphur Powders cleanse and drive out all impurities, and effect an immediate and permanent cure.

Sold by druggists. One package, 12 Powders, $1; six packages, 72 Powders, $5. Mailed free. Hall & Ruckel, 218 Greenwich Street, N. Y.

